# Fast and powerful heritability inference for family-based neuroimaging studies

**DOI:** 10.1016/j.neuroimage.2015.03.005

**Published:** 2015-07-15

**Authors:** Habib Ganjgahi, Anderson M. Winkler, David C. Glahn, John Blangero, Peter Kochunov, Thomas E. Nichols

**Affiliations:** a Department of Statistics, The University of Warwick, Coventry, UK; b Centre for Functional MRI of the Brain, University of Oxford, Oxford, UK; c Department of Psychiatry, Yale University School of Medicine, New Haven, USA; d Olin Neuropsychiatry Research Center, Institute of Living, Hartford Hospital, Hartford, CT, USA; e Department of Genetics, Texas Biomedical Research Institute, San Antonio, TX, USA; f Maryland Psychiatric Research Center, Department of Psychiatry, University of Maryland School of Medicine, Baltimore, MD, USA; g WMG, The University of Warwick, Coventry, UK

**Keywords:** Heritability, Permutation test, Multiple testing problem

## Abstract

Heritability estimation has become an important tool for imaging genetics studies. The large number of voxel- and vertex-wise measurements in imaging genetics studies presents a challenge both in terms of computational intensity and the need to account for elevated false positive risk because of the multiple testing problem. There is a gap in existing tools, as standard neuroimaging software cannot estimate heritability, and yet standard quantitative genetics tools cannot provide essential neuroimaging inferences, like family-wise error corrected voxel-wise or cluster-wiseP-values. Moreover, available heritability tools rely on P-values that can be inaccurate with usual parametric inference methods.

In this work we develop fast estimation and inference procedures for voxel-wise heritability, drawing on recent methodological results that simplify heritability likelihood computations (Blangero etal., 2013). We review the family of score and Wald tests and propose novel inference methods based on explained sum of squares of an auxiliary linear model. To address problems with inaccuracies with the standard results used to find P-values, we propose four different permutation schemes to allow semi-parametric inference (parametric likelihood-based estimation, non-parametric sampling distribution). In total, we evaluate 5 different significance tests for heritability, with either asymptotic parametric or permutation-basedP-value computations. We identify a number of tests that are both computationally efficient and powerful, making them ideal candidates for heritability studies in the massive data setting. We illustrate our method on fractional anisotropy measures in 859 subjects from the Genetics of Brain Structure study.

## Introduction

Combining neuroimaging data with genetic analyses is an increasingly active area of research aimed at improving our understanding of the genetic and environmental control over brain structure and function in health and illness (see, e.g., Glahn etal., 2007). The foundation of any genetic analysis is establishing that a trait is heritable, that is, that a substantial fraction of its variability can be explained by genetic factors. Significant and reproducible heritability has been established for many neuroimaging traits assessing brain structure and function, including, for instance, location and strength of task-related brain activation (Blokland etal., 2008; Koten etal., 2009; Matthews etal., 2007; Polk etal., 2007), white matter integrity (Kochunov etal., 2014a, b; Jahanshad etal., 2013; Brouwer etal., 2010; Chiang etal., 2009, 2011; Kochunov etal., 2010), cortical and subcortical volumes, cortical thickness and density (Winkler etal., 2010; Rimol etal., 2010; Kochunov etal., 2011a, b; Kremen etal., 2010; den Braber etal., 2013).

Variance component models are the best-practice approach for deriving heritability estimates based on familial data (Almasy and Blangero, 1998; Blangero and Almasy, 1997; Amos, 1994; Hopper and Mathews, 1982), for allowing great flexibility in modeling of genetic additive and dominance effects, as well as common and unique environmental influences. The model also allows the inclusion of additional terms that allow linkage analysis, yet remaining relatively simple and requiring the estimation of only a few parameters. Estimation of parameters typically uses maximum likelihood under the assumption that the additive error follows a multivariate normal distribution. The iterative optimization of the likelihood function requires computationally intensive procedures, that are prone to convergence failures, something particularly problematic when fitting data at every voxel/element.

Typically a likelihood ratio test (LRT) is used for heritability hypothesis testing. As the null hypothesis value is on the boundary of the parameter space, the asymptotic distribution of LRT is not *χ*^2^ with 1 degree of freedom (DF), but rather approximately as a 50:50 mixture of *χ*^2^ distributions with 1 and 0 DF, where a 0 DF*χ*^2^ is a point mass at 0 (Chernoff, 1954; Self and Liang, 1987; Stram and Lee, 1994; Dominicus etal., 2006; Verbeke and Molenberghs, 2003). However, this result depends on the assumption of independent and identically distributed (i.i.d.) data (Crainiceanu, 2008; Crainiceanu and Ruppert, 2004a, b, c), which is violated in the heritability problem. It has been shown that 0 values occur at a rate greater than 50%, producing conservative inferences (Blangero etal., 2013; Crainiceanu and Ruppert, 2004a; Shephard, 1993; Shephard and Harvey, 1990).

As with most statistical models, the quantitative genetic models used here are based on an assumption of multivariate Gaussianity, and this assumption is the basis of the estimation and test procedures described above. However, the heritability test statistic's null distribution may be inaccurate even when Gaussianity is perfectly satisfied, due tothe limitations of the 50:50 *χ*^2^ result just mentioned. Further, for neuroimaging spatial statistics, like family-wise error (FWE) corrected inference with either voxel- or cluster-wise inference, the relevant parametric null distributions are intractable. While random field theory (Worsley etal., 1992; Friston etal., 1994; Nichols and Hayasaka, 2003) results exist for *χ*^2^ images (Cao, 1999), they are not directly applicable here as the test statistic image cannot be expressed as a linear combination of component error fields.

Hence, there is a compelling need for alternative inference procedures that make fewer assumptions. Permutation tests are a type of nonparametric test that can provide exact control— or approximately exact when there are nuisance variables— over false positive rates. These tests depend only on minimal assumptions, namely, that under the null hypothesis the data is exchangeable, that is, that the joint distribution of the data remains unaltered after permutation (Nichols and Holmes, 2002; Winkler etal., 2014).

There is relatively little work on permutation tests for variance component inference. The typical application of variance components models is not in quantitative genetics, but in hierarchical linear models where observational units are nested in clusters, such repeated measures designs. Of the few permutation methods proposed in this setting, they all permute the residuals (after removing the covariate effects) between and within clusters while fixing the model structure. While these procedures use different test statistics, e.g. Fitzmaurice and Lipsitz (2007) used the LRT as the statistic, while Lee and Braun (2012) used the sample variance of estimated random effect, they generally require iterative optimization of the likelihood function, and thus as permutation procedures they are yet more computationally demanding.

Samuh etal. (2012) presented a fast permutation test, though it is only applicable to the random intercept model. And recently Drikvandi etal. (2013) introduced a fast permutation test based on the variance least square estimator, which in essence fits a regression model to squared residuals. However, this approach is not based on maximum likelihood, and is only intended for a standard repeated measures model, where independent subjects are recorded multiple times, not multiple dependent subjects as in a pedigree study.

Our group presented a method to accelerate maximum likelihood estimation by applying an orthonormal data transformation that diagonalizes the phenotypic covariance, transforming a correlated heritability model into an independent but heterogeneous variance model (Blangero etal., 2013). However, this advance doesn't eliminate iterative optimization nor possible convergence problems.

In the present work, we expanded upon this work to derive approximate, non-iterative estimates and test statistics based on the first iteration of Newton's method. These procedures can be constructed with an auxiliary model based on regressing squared residuals on the kinship matrix eigenvalues. Then the Wald and score hypothesis tests can then be seen as generalized and ordinary explained sum of squares of the auxiliary model. In addition, as the null hypothesis of no heritability corresponds to homogeneous variance of the transformed phenotype, we draw from the statistical literature on tests of heteroscedasticity for a new and completely different test for heritability detection. We develop permutation test procedures for each of these methods, thus providing FWE-corrected voxel- and cluster-wise inferences.

The remainder of this paper is organized as follows. In the next section we detail the statistical model used and describe each of our proposed methods. The simulation framework used to evaluate the methods, and the real data analysis used for illustration are described in the Evaluation section. We then present and interpret results, and offer concluding remarks.

## Theory

In this section we detail the statistical models used, introduce our fast heritability estimators and tests, and then propose several permutation strategies for these tests.

### Original and eigensimplified polygenic models

At each voxel/element, a polygenic model for the phenotype *Y* measured on *N* individuals can be written as

(1) Y=Xβ+g+ϵ

where *X* is an *N* × *p* matrix consisting of an intercept and covariates, like ageand sex; *β* is the *p*-vector of regression coefficients; *g* is the *N*-vector of latent (unobserved) additive genetic effect; and *ϵ* is the *N*-vector of residual errors. In this study we consider the most common variance components model, with only additive and unique environmental components.

The trait covariance, *Var*(*Y*) = *Var*(*g* + ϵ) = *Σ* can be written as

(2) $$\Sigma =2\sigma_{A}^{2}\Phi +\sigma_{E}^{2}I\text{,}$$

where Φ is the kinship matrix; *σ*_*A*_^2^ and *σ*_*E*_^2^ are the additive genetic and the environmental variance components, respectively; and *I* is the identity matrix. The kinship matrix is comprised of kinship coefficients, half the expected proportion of genetic material shared between each pair of individuals (Lange, 2003).

The narrow sense heritability is

(3) $$h^{2}=\frac{\sigma_{A}^{2}}{\sigma_{A}^{2}+\sigma_{E}^{2}}\text{.}$$

Maximum likelihood is used for parameter estimation with the assumption that the data follows a multivariate normal distribution. The log likelihood for the untransformed model (Eqs. (1)& (2)) is

(4) $$\ell \left(\beta ,\Sigma ;Y,X\right)=-\frac{1}{2}\mathrm{Nlog}\left(2\pi \right)-\frac{1}{2}\log\left(\left|\Sigma \right|\right)-\frac{1}{2}{\left(Y-X\beta \right)}^{\prime }\Sigma^{-1}\left(Y-X\beta \right)\text{.}$$

For large datasets with arbitrary family structure, the computational burden of evaluating of the likelihood can be substantial. In particular, a quadratic form of the inverse covariance, Σ^−1^, must be computed, along with the determinant of Σ. We take the approach of Blangero etal. (2013), who proposed an orthogonal transformation based on the eigenvectors of the kinship matrix, thus diagonalizing the covariance and simplifying the computation of the likelihood (Eq.(4)).

The eigensimplified polygenic model is obtained by transforming the data and model with a matrix *S*, the matrix of eigenvectors of Φ which are the same as the eigenvectors of Σ, Eq. (2). Applying this transformation to Eq. (1) gives the transformed model

$$S^{\prime }Y=S^{\prime }X\beta +S^{\prime }g+S^{\prime }\varepsilon$$

which we write as

(5) $$Y^{*}=X^{*}\beta +\varepsilon^{*}\text{,}$$

where Y* is the transformed data, X* are the transformed covariates and ε* is the transformed random component, where ε* now encompasses both the genetic and non-genetic random variation. The diagonalizing property of the eigenvectors then gives a simplified form for the variance:

(6) $$Var\left(\varepsilon^{*}\right)=\Sigma^{*}=\sigma_{A}^{2}D_{g}+\sigma_{E}^{2}I\text{,}$$

where Σ* is the variance of the transformed data and *D*_*g*_ = diag{*λ*_*gi*_} is a diagonal matrix of the eigenvalues of 2Φ.

The log likelihood takes on the exact same form as Eq. (4) for Y*, X*, *β* and Σ*, except is much easier to work with since Σ* is diagonal:

$$\ell \left(\beta^{*},\sigma_{A}^{*},\sigma_{E}^{*};Y^{*},X^{*}\right)=-\frac{1}{2}\mathrm{Nlog}\left(2\pi \right)-\frac{1}{2}\sum_{i=1}^{N}\log\left(\sigma_{A}^{2}\lambda_{gi}+\sigma_{E}^{2}\right)-\frac{1}{2}\sum_{i=1}^{N}\frac{{\varepsilon_{i}^{*}}^{2}}{\sigma_{A}^{2}\lambda_{gi}+\sigma_{E}^{2}}\text{.}$$

Note that, while *S*′ can be seen as a semi-whitening step, the transformed model can also be seen as a change of variables, where the variance is reparametrized as *Σ* = SΣ*S′. As a reparametrization, the invariance property of maximum likelihood guarantees that the same values of *β*, *σ*_A_^2^ and *σ*_E_^2^ optimize both the original and transformed likelihoods.

Use of this transformation has two major benefits. First, optimization time is substantially reduced, as the inverse and determinant of the transformed covariance are now trivial. Second, applying standard statistical inference procedures, including the score and the Wald test, to the eigensimplified polygenic model produces simple algebraic forms that can be harnessed for fast approximations. Both of these speed improvements facilitate the use of permutation tests that avoid asymptotic approximations.

### Heritability estimation and test statistics

We segregate the transformed model parameters into fixed *β* and random *θ* = (*σ*_A_^2^, *σ*_E_^2^) terms, and estimate them by maximizing the likelihood function via iterative numerical methods. Here, we consider Newton's method because it leads to computationally efficient heritability estimators and associated tests. Newton's method requires the score and expected information matrix of the transformed model, which are

(7) $$S\left(\beta ,\theta \right)=\left[\begin{array}{c} X^{*}\prime \Sigma^{*-1}\varepsilon^{*}\\ -\frac{1}{2}\left[U\prime \Sigma^{*-1}1-U\prime \Sigma^{*-2}\varepsilon^{*2}\right] \end{array}\right]$$

and

(8) $$I\left(\beta ,\theta \right)=\left[\begin{array}{cc} X^{*}\prime \Sigma^{*-1}X^{*} & 0\\ 0 & \frac{1}{2}U\prime \Sigma^{*-2}U \end{array}\right]\text{,}$$

respectively, where *U* = [**1**, *λ*_*g*_] is a *N* × 2 matrix, **1** is a *N* × 1 vector ofones and *λ*_*g*_ = {*λ*_*gi*_} is a *N* × 1 vector of kinship matrix eigenvalues. It is useful to write f* for the vector with elements $f_{i}^{*}={\hat{\varepsilon }}_{i}^{*2}$, where ${\hat{\varepsilon }}^{*}=Y^{*}-X^{*}\hat{\beta }$ are the transformed model residuals. Newton's method gives update equations for $\hat{\beta }$ and $\hat{\theta }$ at iteration *j* + 1 as:

(9) $${\hat{\beta }}_{j+1}={\left(X^{*}\prime {\left({\hat{\Sigma }}_{j}^{*}\right)}^{-1}X^{*}\right)}^{-1}X^{*}\prime {\left({\hat{\Sigma }}_{j}^{*}\right)}^{-1}Y^{*}$$

(10) $${\hat{\theta }}_{j+1}=\max\left\{0,{\left(U^{\prime }{\left({\hat{\Sigma }}_{j}^{*2}\right)}^{-1}U\right)}^{-1}U^{\prime }{\left({\hat{\Sigma }}_{j}^{*2}\right)}^{-1}f_{j}^{*}\right\}\text{,}$$

where *j* indexes iteration; the variance parameters *θ* must be positive, hence the maximum operator. When these updates are iterated until convergence as usual, we denote the estimates with a ML subscript, e.g. ${\hat{\beta }}_{\mathrm{ML}}$, ${\hat{\theta }}_{\mathrm{ML}}$ and ${\hat{h}}_{\mathrm{ML}}^{2}={\hat{\sigma }}_{A,\mathrm{ML}}^{2}\;/\;\left({\hat{\sigma }}_{A,\mathrm{ML}}^{2}+{\hat{\sigma }}_{E,\mathrm{ML}}^{2}\right)$.

To allow for potential improvements on speed, we also consider a one-step estimator. First, observe that since Σ* is diagonal, Eq.(9) is the Weighted Least Squares (WLS) regression of Y* on X*, and Eq.(10) is based on the WLS regression of f* on *U*. This immediately suggests initial values based on Ordinary Least Squares (OLS),

$${\hat{\beta }}_{OLS}={\left(X^{*}\prime X^{*}\right)}^{-1}X^{*}\prime Y^{*}$$

(11) $${\hat{\theta }}_{OLS}=\max\left\{0,{\left(U\prime U\right)}^{-1}U\prime f_{OLS}^{*}\right\}\text{,}$$

where *f*_OLS_^⁎^ is the square of the OLS residuals

(12) $${\hat{\varepsilon }}_{OLS}=Y^{*}-X^{*}{\hat{\beta }}_{OLS}\text{;}$$

while not recommended as a final estimate, it also produces ${\hat{h}}_{OLS}^{2}={\hat{\sigma }}_{A,OLS}^{2}/\left({\hat{\sigma }}_{A,OLS}^{2}+{\hat{\sigma }}_{E,OLS}^{2}\right)$. Finally, our proposed one-step estimators are:

$${\hat{\beta }}_{WLS}={\left(X^{*}\prime {\left({\hat{\Sigma }}_{OLS}^{*}\right)}^{-1}X^{*}\right)}^{-1}X^{*}\prime {\left({\hat{\Sigma }}_{OLS}^{*}\right)}^{-1}Y^{*}$$

(13) $${\hat{\theta }}_{WLS}=\max\left\{0,{\left(U^{\prime }{\left({\hat{\Sigma }}_{OLS}^{*2}\right)}^{-1}U\right)}^{-1}U^{\prime }{\left({\hat{\Sigma }}_{OLS}^{*2}\right)}^{-1}f_{OLS}^{*}\right\}\text{,}$$

where ${\hat{\Sigma }}_{OLS}^{*}$ is formed by ${\hat{\theta }}_{OLS}=\left(\sigma_{A,OLS}^{2},\sigma_{E,OLS}^{2}\right)$, also producing ${\hat{h}}_{WLS}^{2}={\hat{\sigma }}_{A,WLS}^{2}/\left({\hat{\sigma }}_{A,WLS}^{2}+{\hat{\sigma }}_{E,WLS}^{2}\right)$.

Amemiya (1977) showed that such one-step maximum likelihood estimators are asymptotically normal and consistent. Going forward, we will use “ML” to refer to the maximum-likelihood, iterated estimator and “WLS” to refer to this one-step estimator.

### Test statistics

In this section we describe likelihood ratio tests (LRTs), Wald tests, and score test for hypothesis tests of nonzero heritability; we also add an additional test based on detecting heterogeneous variance structure to detect non-zero heritability. We only consider the transformed model, and tests on *H*_0_ : *σ*_A_^2^ = 0 vs. *H*_1_ : *σ*_A_^2^ > 0, equivalent to inference for heritability (Eq.(3)). Table 1 organizes the models and test statistics we consider.

#### Likelihood ratio test

The LRT (Neyman and Pearson, 1933) statistic is twice the difference of the log-likelihoods, unrestricted minus *H*_0_-restricted. For ML this requires optimizing the likelihood function twice, once under the null *H*_0_ : *σ*_A_^2^ = 0, and once under the alternative (though the null model is trivial, equivalent to OLS). We denote the test statistic for this test *T*_L,ML_. In addition, a LRT can be constructed for the transformed model in terms of the one-step WLS estimator; we denote this statistic as *T*_L,WLS_.

#### Wald test

The Wald test consists of a quadratic form of the parameter estimate (minus its null value) and its inverse asymptotic variance (i.e. expected Fisher's information matrix). Both the estimate and its variance are computed under the full, alternative model.

The Wald test for the ML estimator (Rao, 2008) is

$$T_{W,\mathrm{ML}}=\frac{1}{2}{\left({\hat{\sigma }}_{A,\mathrm{ML}}^{2}\right)}^{2}{\left[C{\left(U^{\prime }{\hat{\Sigma }}_{\mathrm{ML}}^{*-2}U\right)}^{-1}C^{\prime }\right]}^{-1}$$

$$=\frac{1}{2}\left(N-{\left(1'{\hat{\Sigma }}_{\mathrm{ML}}^{*-1}1\right)}^{2}{\left(1'{\hat{\Sigma }}_{\mathrm{ML}}^{*-2}1\right)}^{-1}\right)$$

where *C* = [0 1] is a contrast row vector, and the latter is a simpler form found in Buse (1984). Iterative optimization is required for *T*_W,ML_, though it can be more amenable to compute than LRT because the likelihood function is optimized only once.

The Wald test for our one-stepWLS estimator can be written as

$$T_{W,WLS}=\frac{1}{2}{\left({\hat{\sigma }}_{A,WLS}^{2}\right)}^{2}{\left[C{\left(U^{\prime }{\hat{\Sigma }}_{WLS}^{*-2}U\right)}^{-1}C^{\prime }\right]}^{-1}$$

$$=\frac{1}{2}{\left({\hat{\sigma }}_{A,WLS}^{2}\right)}^{2}\times {\left({\hat{\Sigma }}_{OLS}^{*-1}\lambda_{g}\right)}^{\prime }\left(I-{\hat{\Sigma }}_{OLS}^{*-1}1{\left({\left({\hat{\Sigma }}_{OLS}^{*-1}1\right)}^{\prime }\left({\hat{\Sigma }}_{OLS}^{*-1}1\right)\right)}^{-1}1\prime {\hat{\Sigma }}_{OLS}^{*-1}\right){\hat{\Sigma }}_{OLS}^{*-1}\lambda_{g}\text{.}$$

where the second line shows the computation to be half the generalized explained sum of squares (Buse, 1973, 1979) of an auxiliary model, the weighted least squares regression of *f*_OLS_^⁎^ on *λ*_*g*_, with weights determined by ${\hat{\Sigma }}_{OLS}^{*}$.

#### Score test

The score test (Rao, 2008), also known as the Lagrange multiplier test, is a quadratic form of the score (the gradient of the log likelihood) and the expected Fisher's information, each evaluated under the null hypothesis. Among the tests that we consider, the score test is the least computationally demanding procedure, as it only requires estimation of the null model. For *H*_0_ : *σ*_A_^2^ = 0, the score test with the transformed likelihood function is:

$$T_{S}=\frac{\lambda_{g}^{\prime }\Sigma_{OLS}^{*-2}f_{OLS}^{*}-\lambda_{g}^{\prime }\Sigma_{OLS}^{*-1}1}{CU^{\prime }\Sigma_{OLS}^{*-2}UC^{\prime }}$$

$$=\frac{1}{2}{\left(\frac{{\hat{\sigma }}_{A,OLS}^{2}}{{\hat{\sigma }}_{OLS}^{2}}\right)}^{2}\lambda_{g}^{\prime }\left(I-\frac{1'1}{N}\right)\lambda_{g}\text{,}$$

where ${\hat{\sigma }}_{OLS}^{2}={\left({\hat{\varepsilon }}_{OLS}\right)}^{\prime }{\hat{\varepsilon }}_{OLS}/N$ is the OLS naive residual variance estimator. Similar to the Wald test, *T*_S_ can be obtained as half the regression sum of squares of an auxiliary model, the (unweighted) regression of $f^{*}/{\hat{\sigma }}_{A,OLS}^{2}$ on *λ*_*g*_. As it only involves the fitted null model, it isn't associated with a WLS or ML estimate.

We note that Wald and score tests for a null hypothesis value lyingon the boundary of parameter space can take a special form (Freedman, 2007; Molenberghs and Verbeke, 2007; Morgan etal., 2007; Verbeke and Molenberghs, 2007; Silvapulle, 1992; Silvapulle and Silvapulle, 1995; Verbeke and Molenberghs, 2003). However, for our model (Eq.(1)), the standard version is appropriate if the score function is positive at the boundary value and otherwise set to zero. As any negative score values are suppressed by our non-negative constrained estimates ${\hat{\theta }}_{OLS}$ (Eq.(11)) and ${\hat{\theta }}_{WLS}$ (Eq.(13)), our tests are implicitly zero when needed, and thus the appropriate Wald and score tests are as given above.

All three of the LRT, Wald, and score test procedures are asymptotically equivalent but have different small-sample performance, which we evaluate below. These tests are considered to follow asymptotically a 50 : 50 mixture of *χ*^2^ distributions with 1 and 0 DF, where 0 a DF*χ*^2^ is a point mass at 0 (Chernoff, 1954; Self and Liang, 1987; Stram and Lee, 1994; Dominicus etal., 2006; Verbeke and Molenberghs, 2003), although it has been shown that 0 values can occur with a higher frequency, and the standard 50:50 result will tend to produce conservative inferences (Blangero etal., 2013; Crainiceanu and Ruppert, 2004a; Shephard, 1993; Shephard and Harvey, 1990).

#### Goldfeld and Quandt (GQ) test

Instead of standard likelihood theory, an alternative approach to heritability hypothesis testing can be derived from tests of heteroscedasticity. This follows for the transformed model, since the null hypothesis of no heritability corresponds to homoscedasticity of the transformed phenotype variance (Var(ε*) = *σ*^2^*I*). Thus, rejection of the hypothesis of homoscedasticity implies a rejection of the hypothesis of zero heritability. One class of such tests requires an explicit, hypothesized form for the heterogeneous variance. Another type called “nonconstructive” does not require such explicit models; one example is the Goldfeld and Quandt (1965) (GQ) test, which we propose as a test for non-zero heritability.

The GQ test partitions observations into 2 groups, A & B, based on a variable that should explain any heterogeneous variance. The test statistic then compares the ratio of OLS residual mean squares:

(14) $$T_{GQ}=\frac{{\hat{\varepsilon }}_{A}^{*\prime }{\hat{\varepsilon }}_{A}^{*}/\left(n_{A}-1\right)}{{\hat{\varepsilon }}_{B}^{*\prime }{\hat{\varepsilon }}_{B}^{*}/\left(n_{B}-1\right)}$$

where subscript A refers to the high variance group, subscript B to low variance group, ${\hat{\varepsilon }}_{A}^{*}$refers to the residuals from regressing elements of Y* in group A on corresponding rows of X*, and likewise for ${\hat{\varepsilon }}_{B}^{*}$, finally, *n*_*A*_ and *n*_*B*_ are the number of observations in each respective group. With Gaussian errors and under a null hypothesis of homoscedasticity, *T*_GQ_ follows a F-distribution with degrees of freedom *ν*_1_ = *n*_*B*_ − *p* and *ν*_2_ = *n*_*A*_ − *p*, where p is the number of columns in X*.

For the transformed data Y*, the kinship eigenvalues order the variance of the observations when *σ*_A_^2^ > 0. Thus we propose to define the two groups based on *λ*_*gi*_ > 1 and *λ*_*gi*_ ≤ 1, where we make use of the fact ∑_*i*_*λ*_*g*_*_i_*/*N* = *trace*(2Φ)/*N* = 1.

This test is only able to detect non-zero heritability and cannot produce estimates of *h*^2^. On the other hand, the parametric null distribution of (Eq.(14)) does not depend on the mixture approximation and large sample properties, and its implementation is straightforward. To our knowledge, this is the first proposed use of a heteroscedasticity test to create an exact (non-asymptotic), non-iterative test of heritability.

### Permutation test for heritability inference

Permutation methods can be used to construct the null sampling distribution which can be used to produce P-values and thresholds. For the model with only additive genetic and environmental variance components, the null hypothesis of no heritability implies fully independent data. Thus, if there were no nuisance variables (*X*), a permutation test could be conducted by freely permuting the data (*Y*). With covariates, we must permute suitable residuals, as detailed below.

To conduct inference on *σ*_A_^2^ in the presence of the nuisance parameters *β* and *σ*_E_^2^, we draw inspiration from various methods for permutation methods for the GLM (Winkler etal., 2014). For example, there are several different permutation schemes when testing a strict subset of all GLM regression parameters. One approach is to permute only the column of interest in the design matrix. This approach, due to Draper and Stoneman (1966) could be restated as isolating the portion of the model affected by the null hypothesis, and then only permuting that portion. This is the motivation for our first permutation strategy (P1), where we repeatedly fit the model, but randomly permute kinship each time.

Another approach is to use the reduced, null hypothesis model to generate residuals, permute these residuals, and use them as surrogate null data to be re-analyzed (Freedman and Lane, 1983). For the GLM, this is the recommended approach (Winkler etal., 2014), and corresponds to an ideal test where nuisance effects are removed from the data, leaving what should be only unstructured data (under the null) ready to be permuted. This is the motivation for permutation scheme (P2).

Finally, another approach to GLM permutation testing is to use the full, alternative hypothesis model to generate residuals, and then use these residuals as surrogate null data to be re-fit (ter Braak, 1992). This approach has the merit of removing all systematic variation from the data before permutation. This is the motivation for our third and fourth strategies (P3 & P4).

#### Partial model permutation (P1)

We implement approach P1 by permuting just the aspect of the model tested by the *H*_0_. For the untransformed model this corresponds to permuting the model's covariance term to be

$$2\sigma_{A}^{2}P\Phi P^{\prime }+\sigma_{E}^{2}I\text{,}$$

where *P* is one of *N* ! possible *N* × *N* permutation matrices. For the transformed model, the permutated covariance takes the form

$$\sigma_{A}^{2}PD_{g}P^{\prime }+\sigma_{E}^{2}I\text{.}$$

#### Null model residual permutation (P2)

For P2 we generate residuals under *H*_0_ : *σ*_*A*_^2^ = 0, i.e. OLS residuals ${\hat{\varepsilon }}_{OLS}$ (Eq.(12)). Then we permute these residuals, and add-back nuisance (fixed) effects to generate new *H*_0_ realizations Ỹ*:

(15) $${\tilde{Y}}^{*}=X^{*}{\hat{\beta }}_{OLS}+P{\hat{\varepsilon }}_{OLS}^{*}\text{,}$$

where the tilde (^~^) accent denotes one of many realizations, which in turn are fit with the model under consideration.

#### Full model residual permutation (P3)

For P3, we start with full model residuals, i.e. either ${\hat{\varepsilon }}_{\mathrm{ML}}$ or ${\hat{\varepsilon }}_{WLS}$, depending on the estimator used. Then we permute these residuals, and add-back nuisance to generate new null hypothesis realizations; e.g.,for WLS:

(16) $${\tilde{Y}}^{*}=X^{*}{\hat{\beta }}_{WLS}+P{\hat{\varepsilon }}_{WLS}^{*}\text{.}$$

and analogously for ML. Again, each realization *Ỹ* is fit to the current model.

#### Full model whitened residual permutation (P4)

P4 is like P3, but we go a step further and create residuals that are whitened before permutation. For example, for WLS:

(17) $${\tilde{Y}}^{*}=P\left({\hat{\Sigma }}^{*-1/2}{\hat{\varepsilon }}_{WLS}^{*}\right)\text{,}$$

and analogously for ML. Again, each realization is fit to the current model.

In total we have introduced five different test procedures and four permutation strategies, summarized in Table 2.

#### Multiple testing correction

Whether inference is conducted voxel-wise or cluster-wise, the use of use of an uncorrected *α* = 5 % level leads to an excess of false positives. False discovery rate correction, controlling the expected proportion of false positives among all detections, is easily applied based on uncorrected P-values alone (Genovese etal., 2002). As uncorrected permutation cluster-wiseP-values require an assumption of stationarity (though see Salimi-Khorshidi etal. (2010)), FDR is generally only applied with voxel-wiseP-values. Familywise error rate (FWE) correction, controlling the chance of one or more false positives across the whole set (family) of tests (Nichols and Hayasaka, 2003) requires the distribution of the maximum statistic, easily computed for either voxels or cluster size with permutation (Nichols and Holmes, 2002).

## Evaluation

### Simulation studies

We conduct various simulation studies to evaluate proposed methods for heritability inference on the transformed model. The first study considers estimator bias and variance for the different methods. The second study measures the accuracy of parametric and permutation inference methods. Finally, the third study evaluates FWE control in an image-wise setting for voxel and cluster-wise inferences.

In all simulations, the response variable is assumed to be *Y* = *Xβ* + *ε* where *ε* follows *N*(0, *Σ*), *Σ* = *h*^2^(2Φ) + (1 − *h*^2^)*I*. The design matrix *X* consists of an intercept, a linear trend vector *X*_1_ and a quadratic vector *X*_2_ between 1 and − 1, with *β* = [0, 0, 10]. Kinship structure *Φ* is based on real pedigrees (each described below), and the simulations considered a range of true heritabilities (*h*^2^ = 0, 0.2, 0.4, 0.6, 0.8).

#### Simulation 1

This simulation evaluates the bias, standard deviation and mean squared error (MSE) of the heritability estimators (ML and WLS). Thepedigrees and sample sizes used are shown in Table 3; we used pedigrees from the 10th Genetics Analysis Workshop (GAW10) (Mac-Cluer etal., 1997) and from the GOBS dataset (described below). Univariate data *Y* was simulated as per the Gaussian model described above, and 10,000 realizations were used.

#### Simulation 2

This simulation assesses the false positive rates for each method, on the basis of both parametric and permutation methods. For this analysis we used 2 pedigrees from the GAW10 dataset with 138 subjects; the small sample size was used to ‘stress test’ the methods. Univariate data *Y* was simulated as per the Gaussian model described above, 10,000 realizations were used, and 500 permutations for each nonparametric procedure. On the basis of Simulations 1 and 2, ‘winner’ tests and a permutation strategy were chosen and fed into the 3rd simulation study.

#### Simulation 3

Image simulations were conducted under the null hypothesis (*h*^2^ = 0) on a 96 × 96 × 20 image that the response variable for each voxel are simulated as described above, smoothed with a Gaussian filter with a Full Width at Half Maximum of 4 mm. To avoid edge effects, larger images were simulated, smoothed and then truncated. For each realization we collected empirical null distributions of maximum statistic and maximum cluster size to compute FWEP-values; we considered different cluster forming thresholds (parametric uncorrected P-value = 0.05, 0.01, 0.005, 0.001). We generated 5000 realizations and used 500 permutations with each synthetic dataset.

### Application in diffusion tensor imaging data

We used data from the Genetics of Brain Structure and Function Study (GOBS) (Olvera etal., 2011; McKay etal., 2014) to perform voxel and cluster-wiseFA heritability inference in healthy subjects. The sample comprised 859 Mexican–American individuals from 73 extended pedigrees (average size 17.2 people, range = 1–247). The sample was 59 % female (351 men/508 women) and had a mean age of 43.2 (SD = 15.0; range = 19–85). All participants provided written informed consent on forms approved by the Institutional Review Boards at the University of Texas Health Science Center San Antonio (UTHSCSA) and Yale University.

Diffusion imaging was performed at the Research Imaging Center, UTHSCSA, on a Siemens 3 T Trio scanner using a multi-channel phased array head coil. Asingle-shot single refocusing spin-echo, echo-planar imaging sequence was used to acquire diffusion-weighted data with a spatial resolution of 1.7 × 1.7 × 3.0 mm. The sequence parameters were: TE/TR = 87/8000 ms, FOV = 200 mm, 55 isotropically distributed diffusion weighted directions, two diffusion weighting values, b = 0 and 700 s/mm^2^ and three b = 0 (non-diffusion-weighted) images.

ENIGMA–DTI protocols for extraction of tract-wise average FA values were used. These protocols are detailed elsewhere (Jahanshad etal., 2013) and are available online http://enigma.ini.usc.edu/protocols/dti-protocols/. Briefly, FA images from HCP subjects were non-linearly registered to the ENIGMA–DTI target brain using FSL's FNIRT(Jahanshad etal., 2013). This target was created as a “minimal deformation target” based on images from the participating studies as previously described (Jahanshad etal., 2013b). The data were then processed using FSL's tract-based spatial statistics (TBSS; http://fsl.fmrib.ox.ac.uk/fsl/fslwiki/TBSS) analytic method (Smith etal., 2006) modified to project individual FA values on the hand-segmented ENIGMA–DTI skeleton mask. The protocol, target brain, ENIGMA–DTI skeleton mask, source code and executables, are all publicly available (http://enigma.ini.usc.edu/ongoing/dti-working-group/). The FA values are normalized across individuals by inverse Gaussian transform (Servin & Stephens, 2007; Allison etal., 1999) to ensure normality assumption. Finally, we analyzed the voxel and cluster-wiseFA values with applying along the ENIGMA skeleton mask. To validate our proposed methods for heritability estimation and inference for imaging data, we applied them on GOBS dataset.

## Results

### Univariate heritability simulation results

#### Simulation 1

**Fig. 1:**
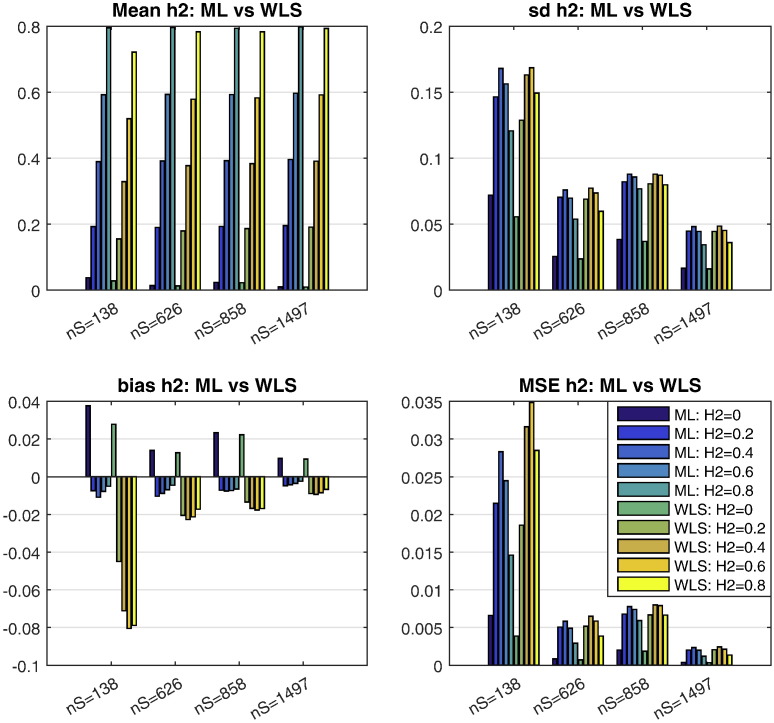
Simulation 1 results, comparing ML and WLS behavior in terms of mean estimate (top left; true *h*^2^ varies on abscissa within clusters), standard deviation (SD; top right), bias (lower left), and mean squared error (MSE; bottom right). See Table 3 for details of each pedigree; nS denotes number of subjects. WLS has worse bias than ML, but small in absolute magnitude, leading to quite similar MSE for large samples.

Fig. 1 compares WLS and ML heritability estimators for various designs and effect sizes, in terms of mean, standard deviation (SD) and mean squared error (MSE), for 10,000 Monte Carlo realizations. Large sample theory dictates that ML should provide best performance, and indeed it has least bias and smallest standard deviation, but the (non-iterative) WLS has MSEs that are only slightly larger. As expected, when the sample size is increased WLS and ML heritability estimators reach almost the same performance. While the WLS estimator bias is worse (more negative) than that of ML, the absolute magnitude of bias is small in large samples.

#### Simulation 2

This simulation assesses the accuracy of parametric null distributions, either a 50:50 *χ*^2^ mixture or *F* distribution, and power. Under *H*_0_, all false positive rates (Table 4) are conservative except *T*_GQ_. The LRT and score tests have Type I error rates that are closer to the nominal level than the Wald tests for the simulated null data (*h*^2^ = 0) but none of them in the MC confidence interval (4.57%–5.42%). Also, the WLS Wald tests had lower error rates than ML Wald tests. In terms of power, the same pattern exists between tests and the LRT and *T*_GQ_ are the most powerful ones.

The conservative false positive rates are attributable to asymptotic null distributions. In particular, the 50:50 mixture approximation has recently been shown to be conservative, which we confirm here. On the other hand, parametric null distribution of *T*_GQ_ does not depend on a mixture approximation and, under a normality assumption, it follows F-distribution exactly; this is likely why GQ had the most accurate false positive rate (4.36%).

**Fig. 2:**
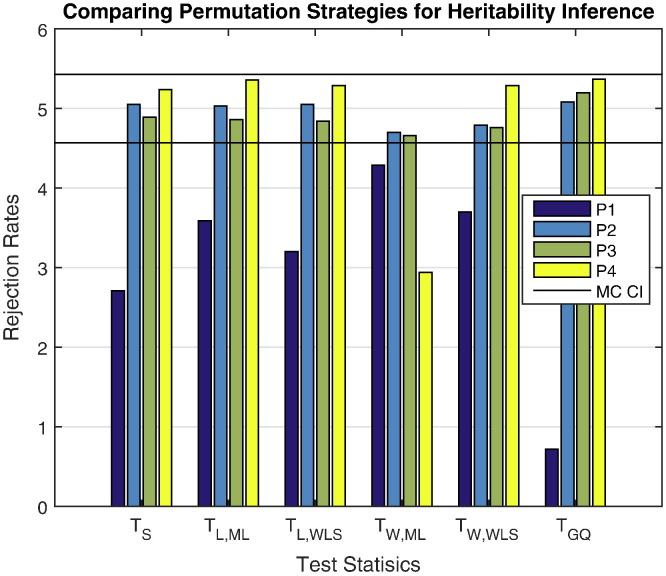
Simulation 2 results, false positive rates for heritability permutation inference, 5% nominal. Based on GAW10 data with 2 families, 138 subjects, 10,000 realizations, and 500 permutations each realization. Monte Carlo confidence interval (MC CI) is (4.57%,5.43%). Permutation schemes P2–P4 generally seem to work well, while *T*_W,ML_ tends to be conservative.

**Fig. 3:**
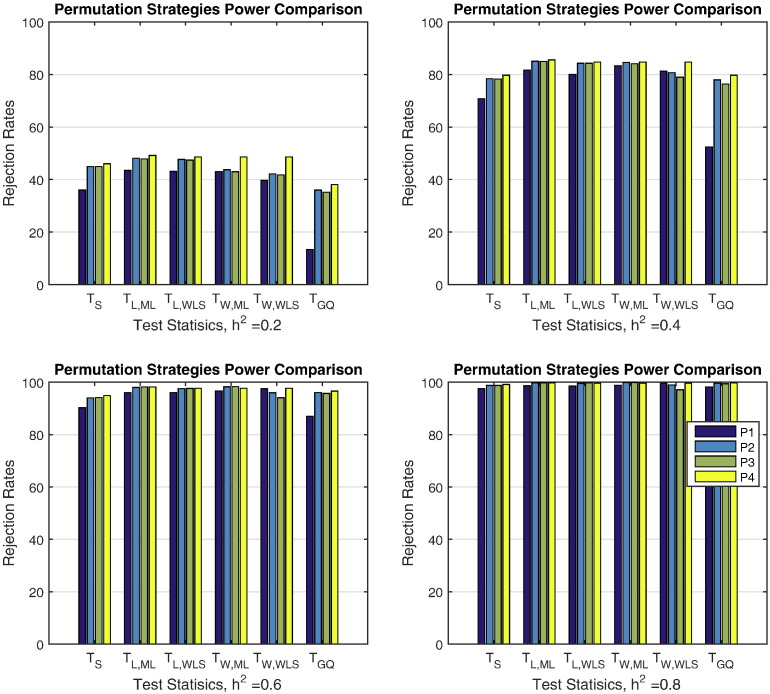
Simulation 2 results, power for heritability permutation inference. For GAW10 data with 2 families, 138 subjects, 10,000 realizations, and 500 permutations each realization. Monte Carlo confidence interval varies with true rejection rate; for 40% it is (39.0%,41.0%), for 80% it is (79.2%,80.8%).

Figs. 2 and 3 show the performance of permutation inference, with rejection rates and power for different effect sizes under the various permutation strategies. Fig. 2 shows that, generally permutation strategy P1 is more conservative than P2, P3 and P4. Moreover the error rates in terms of P2 are close to the nominal level. Although the permutation strategy P4 has higher rejection rates, they still fall within the Monte Carlo confidence interval (4.57%–5.43%) except for *T*_W,ML_.

With respect to power, Fig. 3 shows that again P2, P3 and P4 are generally superior to P1 for various effect sizes. In addition P2, P3 and P4 have almost same performance, all within the Monte Carlo confidence bounds.

Based on all of these results, we selected *T*_S_, *T*_W,WLS_ and *T*_GQ_ and P2 as the computationally most efficient tests to be considered in the image-wise simulations.

### Image-wise simulation results

#### Simulation 3

**Fig. 4:**
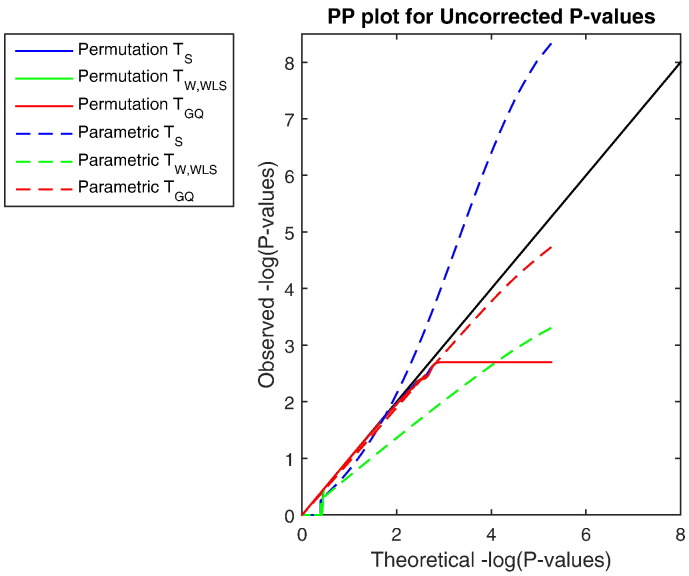
Simulation 3 results, − *log*_10_ PP Plot for uncorrected parametric and permutation P-values for our proposed test statistics. Permutation P-values are valid (solid lines), though are bounded below by 1/500 (above by 2.70 in − *log*_10_*P*), the smallest possible permutation P-value for the 500 permutations used. The permutation P-values are overplotted here, and only the permutation *T*_GQ_ is visible. Parametric P-values for the non-asymptotic GQ test (dashed red line) perform well, while the parametric score test's P-values (dashed blue line) are severely anticonservative (invalid) and Wald test P-values (dashed green line) are severely conservative. Different behavior is seen for P-values larger than 0.5 (smaller than 0.70 in − *log*_10_*P*) as tests giving ≈ 50 % zero values produce ≈ 50 % P-values of 1 (0 in − *log*_10_*P*). Results based on GAW10 data with 2 families, 138 subjects, 5000 realizations, 500 permutations each realization, and 96 × 96 × 20 images with 4 mm FWHM smoothing.

**Fig. 5:**
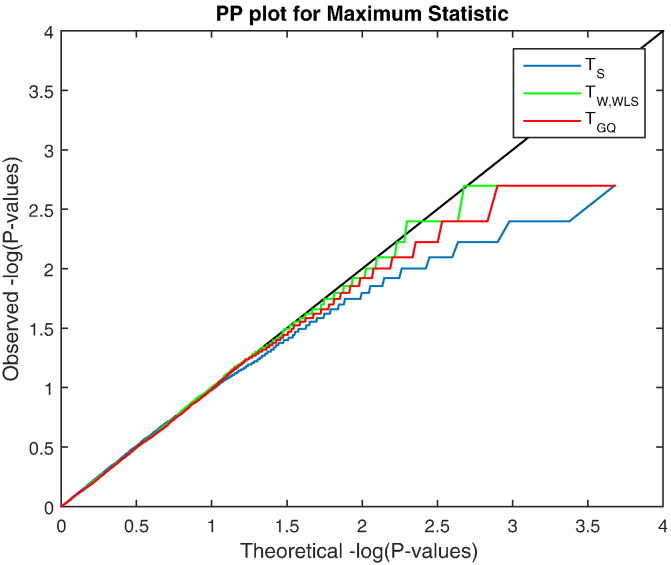
Simulation 3 results, − *log*_10_ PP plot for voxel-wise FWE permutation P-values under the null hypothesis, for three of our proposed test statistics. Each FWE P-value is for the maximum voxel-wise test statistic in each realized dataset. All three test statistics produce valid P-values, though are bounded below by 1/500 (above by 2.70 in − *log*_10_*P*). The Wald test's FWE it slightly conservative, and score a bit more so. Results based on GAW10 data with 2 families, 138 subjects, 5000 realizations, 500 permutations each realization, and 96 × 96 × 20 images with 4 mm FWHM smoothing.

This simulation evaluates false positive rate control in the more challenging image-wise setting, for both voxel and cluster-wise heritability inference. Fig. 4 shows the P–P plot of uncorrected P-values, plotted as− *log*_10_*P*-values. Except for modest conservativeness (*P* ≈ 10^− 2.5^), and of course the truncation due to limited permutations (500 permutations, minimal P-value of 0.002, maximum − *log*_10_*P*-value of 2.69), the accuracy is quite good over-all. Fig. 5 show that FWE-correctedP-values are also accurate, with slight conservativeness with the GQ test. For the 5% level specifically, voxel-wiseFWE for the score, the Wald and the GQ tests were 5.08 %, 5.44 % and 5.4 % respectively, well within the Monte Carlo 95% CI, (4.40%–5.60%).

**Fig. 6:**
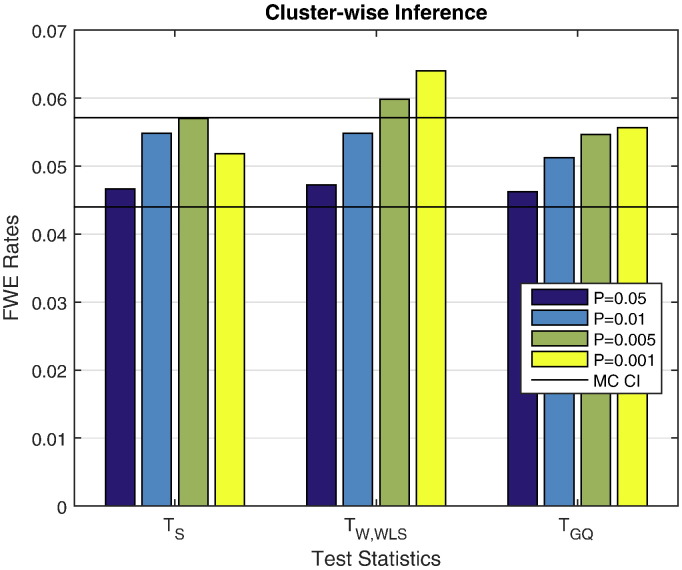
Simulation 3 results, FWE error rates for cluster-wise permutation heritability inference under the null hypothesis, for three of our proposed test statistics. Score and GC test have nominal false positive rates, while the Wald test is anticonservative for high (uncorrected P of 0.005 & 0.001) clustering forming thresholds. This is likely due to use of parametric cluster-forming threshold; see text for more discussion. Results based on GAW10 data with 2 families, 138 subjects, 5000 realizations, 500 permutations each realization. Monte Carlo 95% confidence interval (4.40%,5.60%).

Fig. 6 shows cluster-wiseFWE rates for different cluster forming thresholds. All rates are nominal except for the higher cluster forming thresholds of *T*_W,WLS_(P = 0.005 & P = 0.001). The cluster-forming thresholds come from the parametric null distribution, and Fig. 4 shows severe conservativeness for *T*_W,WLS_'s parametric P-values. For example, that figure shows that when a P = 0.001 uncorrected threshold is used for *T*_W,WLS_, the actual false positive rate is less than 0.0001. This effect, combined with variation of effective false positive rate of the cluster-forming threshold over permutations, could explain this slight anticonservativeness.

**Fig. 7:**
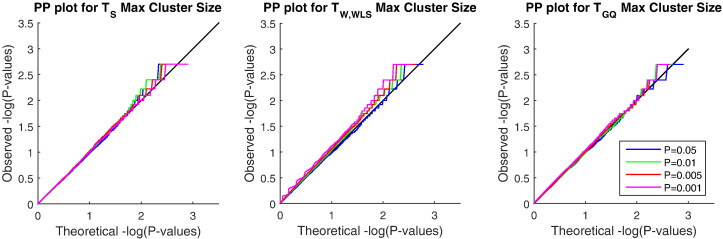
Simulation 3 results, − *log*_10_ PP plots for cluster-wise FWE permutation P-values under the null hypothesis, for three of our proposed test statistics. Each FWE P-value is for the maximum cluster size in each realized dataset. GQ has most accurate FWE P-values, followed by the score test; Wald is slightly anticonservative for high cluster forming thresholds; see text for discussion. For GAW10 data with 2 families, 138 subjects, 5000 realizations, 500 permutations each realization (MC CI = (4.40,5.60)).

Fig. 7 compares the selected test maximum cluster size P-values based on different cluster forming thresholds with their theoretical values; again *T*_W,WLS_behavior for large cluster forming thresholds shows slightly inflated rejection rates.

### Real data analysis

**Fig. 8:**
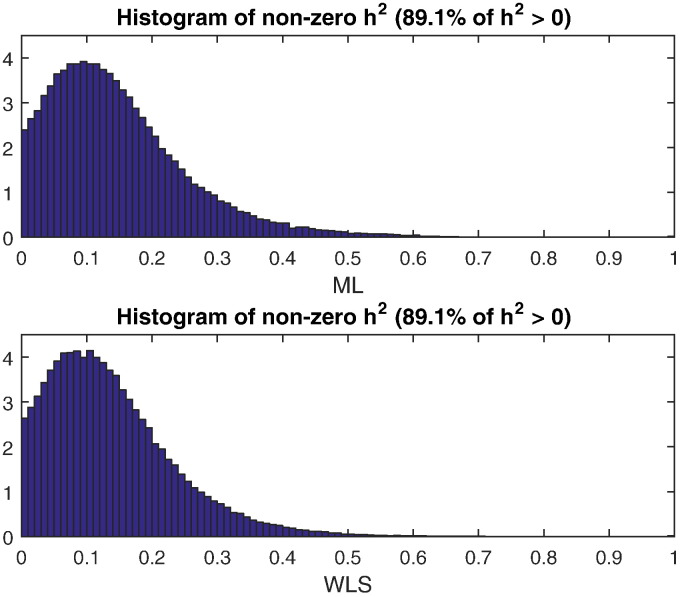
Real data results, comparison of voxel-wise heritability estimates from ML and WLS estimates. The histograms show that the estimates from the two methods are largely similar.

**Fig. 9:**
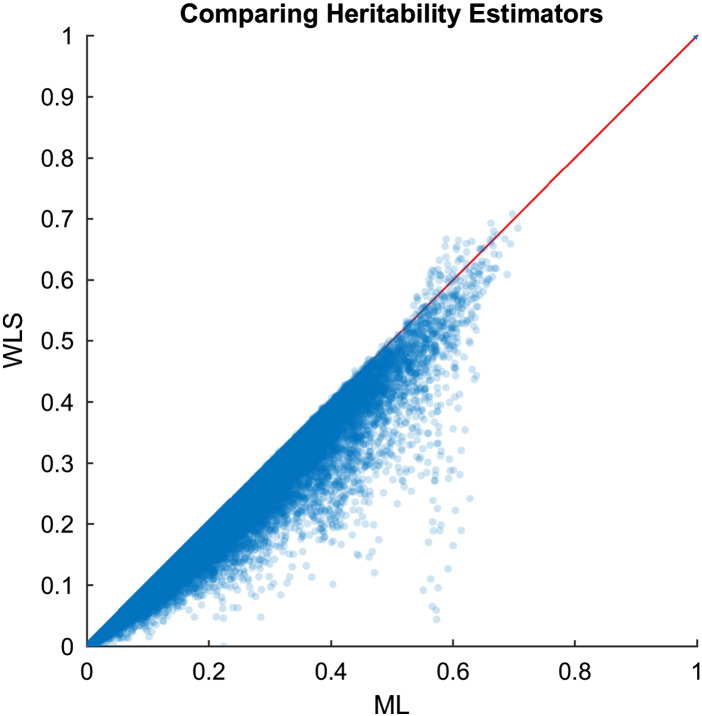
Real data results, scatterplot of voxel-wise heritability estimates from ML and WLS estimates. The two methods are largely similar, though ML is almost always larger than WLS estimates.

**Fig. 10:**
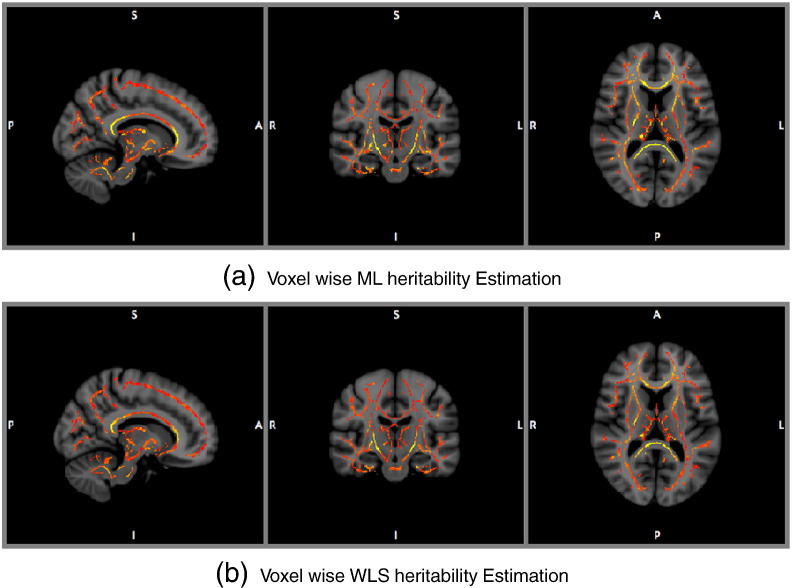
Real data results, voxel-wise heritability estimates for ML (top) and WLS (bottom). Heritability shown in hot-metal color scale, intensity range [0,0.5] for both, overlaid on MNI reference brain. Differences only apparent in highest FA areas.

Voxel-wiseFA heritability estimation and inference for the GOBS study are shown with ML and WLS estimators, creating four test statistic images: *T*_L,ML_, *T*_S_, *T*_W,WLS_, and *T*_GQ_; permutation scheme P2 was used to compute uncorrected and FWE-correctedP-values. Fig. 8 shows histograms of *h*_ML_^2^ (top) and *h*_ML_^2^ (bottom), showing generally the same distribution of heritability over the white matter skeleton. Fig. 10 shows *h*^2^ estimates on the TBSS skeleton. Fig. 9 directly compares WLS and ML heritability estimates with a scatter plot, showing a slight but consistent trend towards underestimation of *h*_ML_^2^ relative to *h*_ML_^2^, consistent with simulation (Fig. 1).

**Fig. 11:**
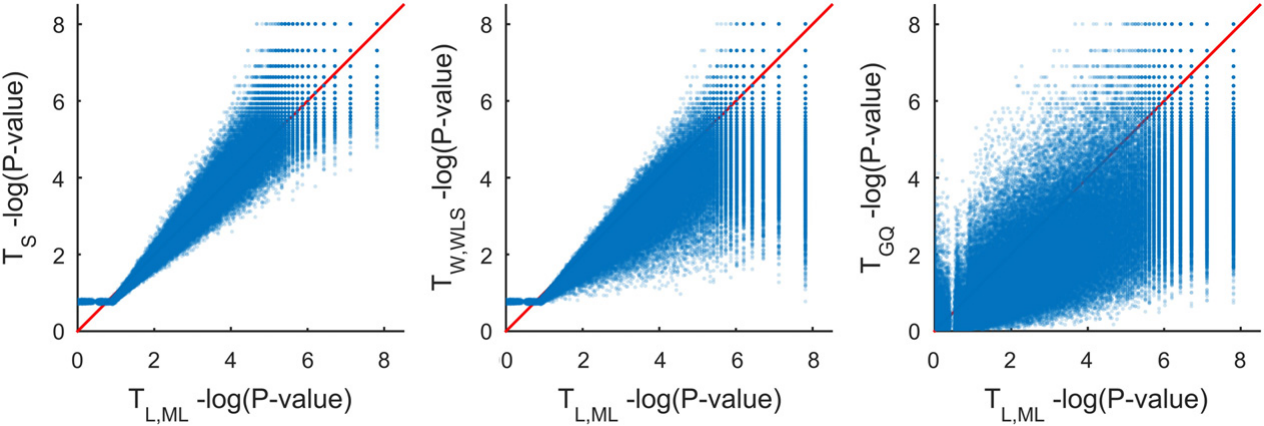
Real data results, scatter plots of voxel-wise uncorrected − *log*_10_*P*-values for score, WLS Wald and GQ tests vs. the ML LRT test. Score P-values are most faithful representation of the ML LRT P-values, while WLS Wald P-values tend to be more conservative; GQ P-values are much more different and generally more conservative.

Voxel-wise uncorrected − *log*_10_P-values from *T*_S_, *T*_W,WLS_, *T*_GQ_ and *T*_L,ML_ based on P2 are compared in Fig. 11. Considering *T*_L,ML_ as a reference (on the abscissa), *T*_W,WLS_ and *T*_GQ_ are generally less sensitive than *T*_L,ML_(Fig. 11 middle and right panels), consistent with the simulations above. However, *T*_S_ was more comparable with *T*_L,ML_(Fig. 11 left panel). Level 5% FWE-corrected statistic thresholds for *T*_S_, *T*_W,WLS_, *T*_L,ML_ and *T*_GQ_ are 39.92, 18.31, 24.27 and 1.72, respectively, producing significant voxel counts of 8521, 1043, 7418 and 2446, respectively, out of 117,139 voxels.

**Fig. 12:**
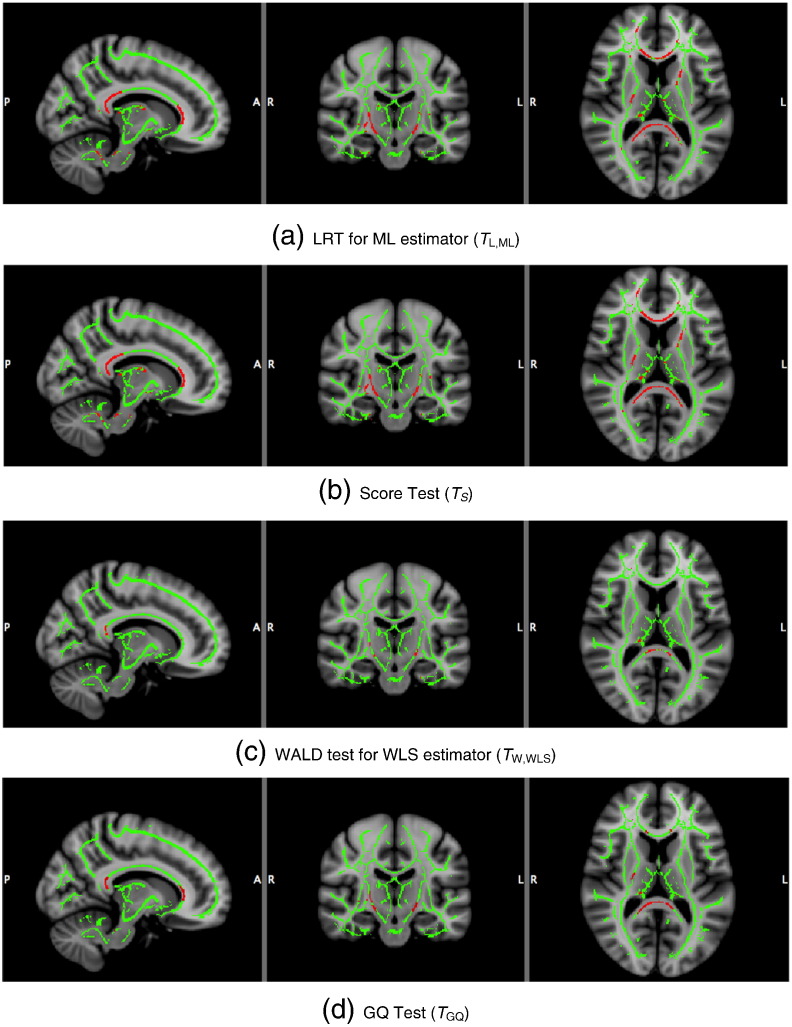
Real data results, voxel-wise 5% FWE significant heritability, for 4 different methods. Full skeleton and significant voxels are in green and red, respectively. The non-iterative score test gives very similar results to the ML (fully iterated) LRT, with the other 2 methods being less sensitive.

**Fig. 13:**
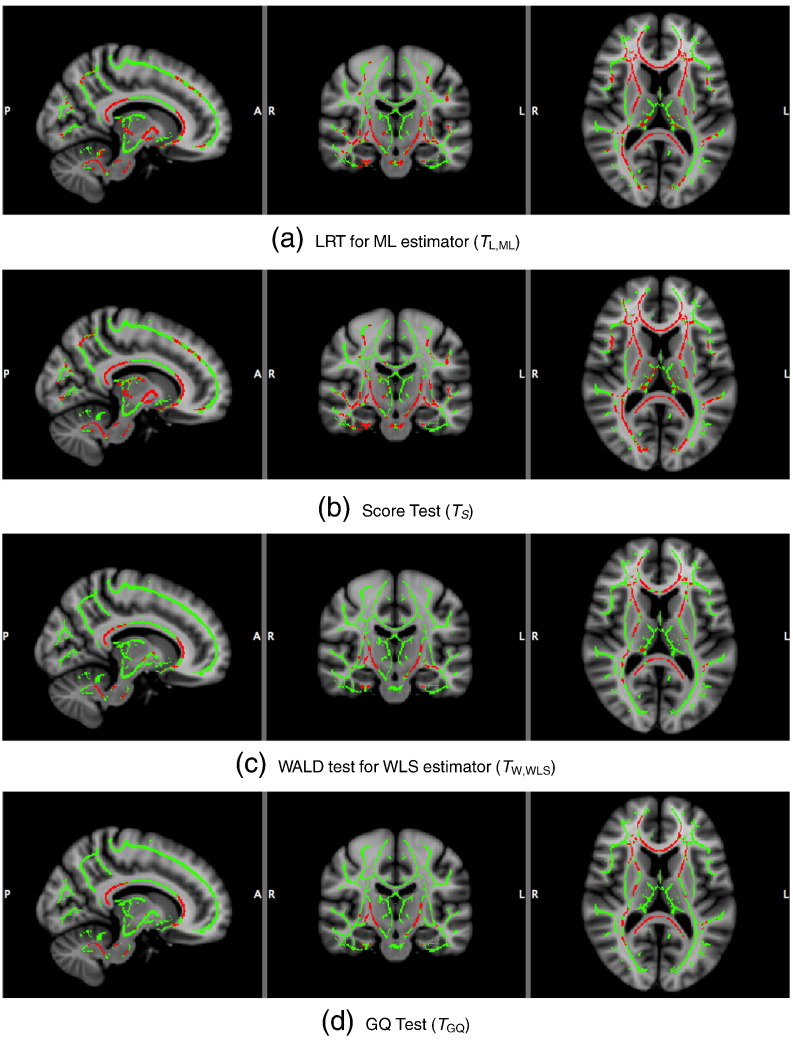
Real data results, cluster-wise 5% FWE significant heritability, for 4 different methods, cluster-forming threshold parametric uncorrected P = 0.01. Full skeleton and significant voxels are in green and red, respectively. Methods appear more similar, but again the non-iterative score test is most similar to the ML LRT result.

Cluster-wise inference results for cluster forming thresholds corresponded to uncorrected P-value = 0.01 % are shown in Table 5 the tests that we consider. Level 5% FWE-corrected cluster size thresholds for *T*_S_, *T*_W,WLS_, *T*_L,ML_ and *T*_GQ_ are 265, 98, 142 and 135 voxels, respectively. For voxel-wise inference, Fig. 12, the score test was most similar to ML's LRT, and likewise for cluster-wise inference, Fig. 13.

## Discussion & conclusions

We have proposed a number of computationally efficient tests for heritability with family data. To our knowledge this is the first work that enables practitioners to study brain phenotype heritability in each voxel without confronting an intense computational burden. Our methods are based on the eigensimplified model of Blangero etal. (2013), most of which can be implemented with auxiliary models, corresponding to regressing squared OLS residuals on the kinship matrix eigenvalues.

For heritability estimation our WLS method, based on one step of Newton's method, was a fast and reasonable approximation to fully iterated ML, ideal for application to brain image data.

For heritability inference, we found that parametric P-values for LRT, Wald and score methods were all conservative, likely due to the untenable i.i.d. assumption underlying the 50:50 *χ*^2^ mixture approximation. As an alternative, permutation test error rates were much closer than parametric one to the nominal level. Notably, all of our simulations included fixed effects covariates (*X*).

The GQ heteroscedasticity test, adapted here for heritability detection, had good performance in simulation, with the best false positive control and respectable power, but on the real data was dramatically different (see Fig. 12) and apparently less powerful.

Image wise simulation results showed that FWE-correctedvoxel- and cluster-wise inference was valid at the 5% level for *T*_S_ and *T*_GQ_, permutation scheme P2. In real data, the P-values for *T*_GQ_ were less similar to the LRT results than the score or Wald test, and was less sensitive over all. The GQ test's power depends on the cut point used to define the two groups, though we did not investigate further. On balance we suggest the use of *T*_S_ for standard neuroimaging inference tool including voxel and cluster-wise inference.

Running time for different test statistics that were presented in Table 6 based on a benchmark with Intel(R)core(TM) i7-2600CPU @ 3.4 GH and 16 GB RAM feature confirms that the empirical null distribution of explained sum of squares of auxiliary model (*T*_S_) under the permutation scheme P2 can be derived substantially faster than *T*_L,ML_, the classic test statistic for heritability inference. Although the sample size plays an important role in running time, we believe that *T*_S_ can be derived significantly faster than the other tests, since it does not depend on numerical optimization. Hence, the whole permutation distribution can be derived easily, either for a univariate trait or a multivariate spatially dependent neuroimaging data accounting explicitly for family wise error.

Finally, we note that yet-more computationally efficient estimates can be obtained by conditioning on the over-all variance estimate, ${\hat{\sigma }}^{2}$, which leads to a 1-parameter variance model. However, in initial simulations we found that this lead to greater bias in *h*^2^ and specifically *h*^2^ estimates in excess of 1.0. Thus we retained the 2-parameter variance model.

In conclusion, our results present a novel inference technique to be implemented in the genetic imaging analysis software like SOLAR-Eclipse(http://www.nitrc.org/projects/se_linux). These methods provide fast inference procedure on millions of phenotypes, filtering a small number of elements for further investigation with more computational intense tools. In future work we will extend these tools for inference on covariates, in particular permutation-based tests for voxel-wiseGWAS analysis for family based data.

The methods in this work will soon be found in the SOLAR and SOLAR-Eclipse packages, and a Matlab implementation is available at http://warwick.ac.uk/tenichols/FPHI.

## Figures and Tables

**Table 1:**
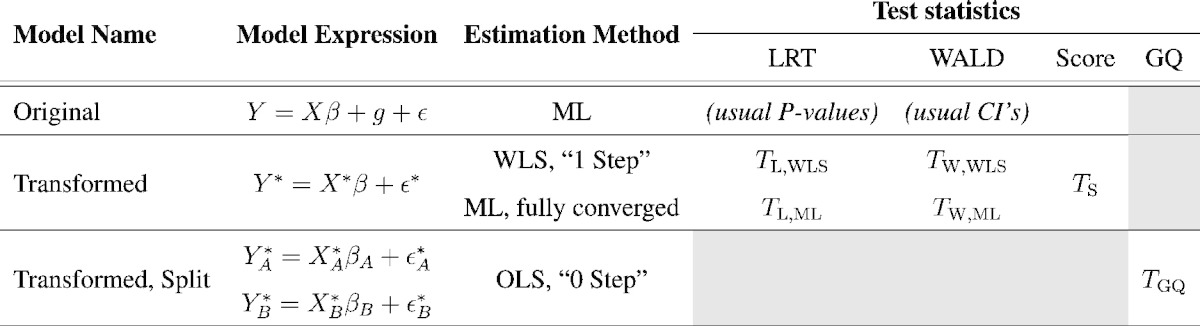
Comparison of model and test statistic properties. Usual P-values and CI's (confidence Intervals) refer to the best practice inference tools used with maximum likelihood estimation.

**Table 2:** Comparison of tests for heritability inference.

Tests	*h*^2^ estimates	Distribution	Type	Optimization	Permutation
*T*_L,ML_		50:50 *χ*_1_^2^ and 0	Asymptotic	ML	P1, P2, P3, P4
*T*_W,ML_		50:50 *χ*_1_^2^ and 0	Asymptotic	ML	P1, P2, P3, P4
*T*_W,WLS_		50:50 *χ*_1_^2^ and 0	Asymptotic	WLS	P1, P2, P3, P4
*T*_S_		50:50 *χ*_1_^2^ and 0	Asymptotic	OLS	P1, P2, P3, P4
*T*_GQ_		$F_{n_{2}-p,n_{1}-p}$	Exact	OLS	P1, P2, P3, P4

Proposed test procedures: The score test (*T*_S_), the Wald test and its variants in terms of WLS estimators (*T*_W,WLS_) and ML estimators (*T*_W,ML_), and the LRTs in terms of the transformed model (*T*_L,ML_). ML optimization denotes iterative optimization until convergence; WLS a 1-step of Newton's method; and OLS an estimate based on (unweighted) least squares.

**Table 3:** Datasets used in simulation 1.

Datasets	Number of pedigrees	Sample size
GAW10	2	138
GAW10	9	626
GOBS	73	858
GAW10	23	1497

**Table 4:** Simulation 2 result, comparing parametric rejection rates (percent), 5% nominal. For GAW10 data with 2 families, 138 subjects, 10,000 realizations. GQ test has the most accurate false positive rate, LRT with ML (*T*_L,ML_) is the most powerful; both GQ (*T*_GQ_) and score (*T*_S_) test have good power (95% MC CI for 0.05, i.e. for the null case is (4.57%, 5.42%)).

Test	True effect (*h*^2^)				
	0	0.2	0.4	0.6	0.8
*T*_S_	3.76	40.66	76.76	94.32	98.94
*T*_W,WLS_	1.56	26.94	73.46	95.62	99.64
*T*_W,ML_	2.50	33.00	77.74	94.84	97.54
*T*_L,ML_	3.16	42.28	81.80	96.40	98.90
*T*_GQ_	4.36	35.60	78.22	96.50	99.70

**Table 5:** Real data results, cluster-wise inferences with different methods.

Method	Total # of clusters	# of significant clusters	Largest cluster size	Smallest corrected P-value
*T*_L,ML_	1770	22	24,246	0.0005
*T*_W,WLS_	1725	19	3643	0.0003
*T*_S_	1689	11	31,250	0.0003
*T*_GQ_	1751	20	4383	0.0003

Cluster-wise inference for *T*_L,ML_, *T*_W,WLS_, *T*_*S*_ and *T*_GQ_. Based on 858 subjects from GOBS and 3000 permutations.

**Table 6:** Computation times. Comparison of running times for a dataset with 138 subjects, 2 families, (GAW10 kinship) and 184,320 voxels. Run on Intel(R)core(TM) i7-2600 CPU @ 3.4 GH and 16 GB RAM.

Statistics	Univariate trait	Image-wise trait
*T*_L,ML_	1 s	8 h
*T*_W,WLS_	0.005 s	2 s
*T*_S_	0.005 s	2 s
*T*_GQ_	0.004 s	1.5 s
